# Optimized Analytical Procedures for the Untargeted Metabolomic Profiling of Human Urine and Plasma by Combining Hydrophilic Interaction (HILIC) and Reverse-Phase Liquid Chromatography (RPLC)–Mass Spectrometry*[Fn FN2]

**DOI:** 10.1074/mcp.M114.046508

**Published:** 2015-03-18

**Authors:** Kévin Contrepois, Lihua Jiang, Michael Snyder

**Affiliations:** From the Department of Genetics, Stanford University School of Medicine, Stanford, CA, USA

## Abstract

Profiling of body fluids is crucial for monitoring and discovering metabolic markers of health and disease and for providing insights into human physiology. Since human urine and plasma each contain an extreme diversity of metabolites, a single liquid chromatographic system when coupled to mass spectrometry (MS) is not sufficient to achieve reasonable metabolome coverage. Hydrophilic interaction liquid chromatography (HILIC) offers complementary information to reverse-phase liquid chromatography (RPLC) by retaining polar metabolites. With the objective of finding the optimal combined chromatographic solution to profile urine and plasma, we systematically investigated the performance of five HILIC columns with different chemistries operated at three different pH (acidic, neutral, basic) and five C18-silica RPLC columns. The zwitterionic column ZIC-HILIC operated at neutral pH provided optimal performance on a large set of hydrophilic metabolites. The RPLC columns Hypersil GOLD and Zorbax SB aq were proven to be best suited for the metabolic profiling of urine and plasma, respectively. Importantly, the optimized HILIC-MS method showed excellent intrabatch peak area reproducibility (CV < 12%) and good long-term interbatch (40 days) peak area reproducibility (CV < 22%) that were similar to those of RPLC-MS procedures. Finally, combining the optimal HILIC- and RPLC-MS approaches greatly expanded metabolome coverage with 44% and 108% new metabolic features detected compared with RPLC-MS alone for urine and plasma, respectively. The proposed combined LC-MS approaches improve the comprehensiveness of global metabolic profiling of body fluids and thus are valuable for monitoring and discovering metabolic changes associated with health and disease in clinical research studies.

Metabolomics is a relatively recent “omic” that aims at measuring the quantity of a large collection of metabolites (*i.e.* low-molecular-weight organic compounds, typically < 1,500 Da). It is often applied to the study of human diseases (1, 2) (*i.e.* characterization of deregulated metabolic pathways and discovery of therapeutic targets and biomarkers), drug toxicity and efficacy (3), and environmental exposure (*e.g.* food (4, 5)) and lifestyle (*e.g.* fitness (6)) on health. Metabolomics is advantageous over other “omics” (*i.e.* genomics, transcriptomics, and proteomics) because it measures a more direct functional readout of activity and phenotype (7). When applied to biofluids (*i.e.* urine and blood), the profiling of metabolites reveals a snapshot of the “metabolic status” of the subject and as such holds great promise for personalized metabolomics and medicine (8, 9).

Metabolic profiling studies are mostly performed using i) chromatography coupled to mass spectrometry (MS) instruments, including gas chromatography (GC)-MS and liquid chromatography (LC)-MS, as well as ii) nuclear magnetic resonance (NMR) spectroscopy platforms. Few studies have highlighted the benefit of combining multiplatform approaches for the analysis of urine and blood (10–12). However, due to instrumentation limitation, most laboratories use a single analytical approach. Because of its high sensitivity and wide range of metabolites that can be analyzed, LC-MS utilization has expanded rapidly over the past 10 years (13). Most untargeted studies are performed using reverse-phase liquid chromatography (RPLC, mainly C18-bonded silica columns) because it generates reproducible data for a large set of metabolites (non- and moderately polar compounds) (14, 15). However, many metabolites in biofluids are water soluble, polar, and ionic (*e.g.* amino acids, organic acids, sulfates, and sugars) and are usually not retained on RPLC columns, thus hindering their identification and accurate quantification (16, 17).

Hydrophilic interaction liquid chromatography (HILIC)^1^ is now becoming popular as it offers a complementary selectivity to RPLC (18–21). A plethora of HILIC stationary phases have been developed and can be separated in four categories: i) anionic (mostly bare silica), ii) cationic (silica derivatized with a positively charged chemical group, mostly aminopropyl), iii) uncharged (silica derivatized with an uncharged chemical group, mostly amide), and iv) zwitterionic (silica derivatized with a chemical group bearing a positive and a negative charge, mostly sulfobetaine). The different HILIC stationary phases and their use have been extensively reviewed (22–24). HILIC methodologies have mostly been optimized for targeted analyses focusing on a small subset of metabolites (*i.e.* nucleosides and derivatives (25), neurotransmitters (26), and peptides (27)). Despite its usefulness for targeted analyses, HILIC-MS still represents a challenge in untargeted metabolic profiling studies because it is less reproducible (retention time and MS signal drift with time) and requires longer equilibration time than RPLC-MS (19, 20). As such, less than 15% of the LC-MS-based untargeted metabolomic studies performed on biofluids published in 2013 used both HILIC- and RPLC-MS (28–32). Among these studies, there was no consensus on the analytical procedure (column and chromatographic conditions) that will produce the optimal reproducibility and metabolome coverage for the global analysis of urine and blood.

In this context, we systematically investigated the performance of a variety of HILIC and RPLC columns under different chromatographic conditions using standard mixtures and biological samples. After having determined the optimal chromatographic solution for profiling metabolites from urine and plasma using HILIC- and RPLC-MS, we estimated the intra- and long-term (40 days) interbatch reproducibility of retention time and peak area of the optimized LC conditions. Adequate equilibration and conditioning of the HILIC column resulted in excellent reproducibility similar to RPLC-MS approaches. Finally, we investigated the complementarity of HILIC and RPLC in positive and negative electrospray ionization (ESI) modes and found that the optimized HILIC condition greatly expanded the metabolome coverage with 44% and 108% new metabolic features detected, compared with RPLC-MS alone in urine and plasma, respectively.

## EXPERIMENTAL SECTION

### 

#### 

##### Chemical Material and Standard Sample Preparation

Analytical grade standards were purchased from various companies as detailed in the supplementary Excel file S1. Four standard mixtures containing a total of 174 diverse compounds were prepared for HILIC-MS at a final concentration of 10 μm in 50% acetonitrile. For RPLC, one standard mixture containing 22 compounds was prepared at a final concentration of 10 μm in 5% acetonitrile. Ammonium acetate, ammonium hydroxide, formic acid, and acetic acid were purchased from Sigma Aldrich (St. Louis, MO, USA). MS-grade water, acetonitrile, and methanol were from Fischer Scientific (Morris Plains, NJ, USA).

##### Biological Material and Sample Preparation

Residual blood and urine samples were obtained from de-identified donors under informed consent approved by Stanford's Institutional Review Board. After collection, urine samples were immediately centrifuged at 21,000g for 10 min at 4 °C. The supernatant was aliquoted and stored at −80 °C prior to analysis. Urine samples were diluted by a factor of four with 75% acetonitrile and 100% water for HILIC- and RPLC-MS experiments, respectively. Blood was obtained from the Stanford Blood Center. Plasma was prepared from whole blood treated with anti-clot EDTA and kept at 4 °C for 2 h before being aliquoted and stored at −80 °C. Plasma was treated with four volumes of a acetone:acetonitrile:methanol (1:1:1, v/v) solvent mixture, mixed for 15 min at 4 °C and incubated for 2 h at −20 °C to allow protein precipitation (8). The supernatant was collected after centrifugation at 10,000 rpm for 10 min at 4 °C and evaporated to dryness. The dry extracts were reconstituted with 50% methanol before analysis.

##### Instrumentation and LC-MS Acquisitions

Standard mixtures, urine and plasma samples were analyzed using an Agilent 1260 Infinity HPLC system coupled to an Agilent 6538 UHD Q-TOF MS. The Q-TOF was equipped with an ESI probe and operated in positive and negative modes using the MS full scan mode. The data were acquired between 50 and 1,000 *m/z* at a scan rate of 1.5 spectra/s in centroid mode at a resolution of 10,000 (*m/z* 127). The source conditions were as follow: gas temperature 325 °C, drying gas 9 l/min, nebulizer 45 psig, fragmentor 125 V, skimmer 47 V and capillary voltage 3,500V or −3,500V in positive or negative ESI modes, respectively. Reference masses 121.0509 (Purine) and 922.0098 (hexakis(1H, 1H, 3H-tetrafluoropropoxy)phosphazine, HP-0921) in positive mode and 112.9856 (TFA anion) and 980.0164 (HP-0921 + acetate) in negative mode were used for internal mass calibration during the runs.

##### Chromatographic Conditions

Five HILIC columns were compared: BEH (Ethylene Bridged Hybrid) amide, BEH HILIC, Hypersil GOLD HILIC, Syncronis HILIC, ZIC-HILIC (see the supplementary experimental section for more details). Mobile phases for HILIC at neutral pH 6.9 consisted of 10 mm ammonium acetate in 5/95 acetonitrile/water (A) and 10 mm ammonium acetate in 95/5 acetonitrile/water (B). Mobile phases A and B were modified with 0.1% formic acid for acidic pH 3.4 conditions and with 0.5% of 25% ammonium hydroxide for basic pH 10.15 conditions. The pH was measured in absence of acetonitrile. Metabolites were eluted from the columns at 0.5 ml/min using a 1–50% phase A gradient over 15 min. Before each injection, the column was equilibrated for 5 min with 1% phase A. The oven temperature was set to 40 °C, and the injection volume was 5 μl. Five RPLC columns were compared: Hypersil GOLD, Hypersil GOLD aq, BEH C18, Kinetex, and Zorbax SB (see the supplementary experimental section for more details). Mobile phases for RPLC consisted of 0.06% acetic acid in water (A) and MeOH containing 0.06% acetic acid (B). Metabolites were eluted from the column at a flow rate leading to a backpressure of 260–280 bar at 99% phase A. A 1–80% phase B gradient was applied over 9–10 min (see the supplementary experimental section). The oven temperature was set to 60 °C and the injection volume was 5 μl.

##### Data Processing and Analysis

Metabolic features (characterized by a unique mass/charge ratio and retention time) were extracted with the MassHunter Qualitative Analysis Software B.05.00 (Agilent Technologies, Santa Clara, CA) with an absolute height filter set to 10,000 counts. A scoring system was created to assign a score to standards and metabolic features from biological samples. The scoring system takes into account the retention time, peak shape, and MS signal (see the supplementary experimental section for details). When the score was not calculated, metabolic features were extracted with the XCMS (various forms (X) of chromatography mass spectrometry) (33) package (version 1.39.4) in R (version 3.0.1) with an absolute height filter set to 2,000 counts. Grouping and annotation were performed with the CAMERA (34) package (version 1.16.0). The parameters used in XCMS and CAMERA are detailed in the supplementary experimental section. Features were putatively identified by matching the accurate masses (± 5 ppm) against a local database containing 33,442 entries generated by Creek *et al.*, (35) with slight modifications.

## RESULTS

### Optimization of the HILIC-MS Analytical Procedure

Five different HILIC columns were examined; two columns were composed of uncharged stationary phases (BEH amide and Hypersil GOLD HILIC), one column was anionic (BEH HILIC), and two were zwitterionic (Syncronis HILIC and ZIC-HILIC). Bare silica, amide, and zwitterionic columns were selected for their complementarity of selectivity toward a wide variety of compounds (26, 27, 36, 37). We did not assay the performance of a cationic column containing an aminopropyl group (such as Luna NH_2_) due to its reported shorter life time (22, 28, 38). The tested columns were operated at three different pH conditions: i) acidic (pH 3.4), ii) neutral (pH 6.9), and iii) basic (pH 10.15). Only the BEH amide column was subjected to basic pH because the stationary phases of the other columns were not stable above pH 8–9. Altogether, 11 chromatographic conditions were run in both positive and negative ESI modes and compared using standards commonly found in biofluids and human urine samples.

#### 

##### Performance of the HILIC Conditions Using Standards

**Fig. 1.**
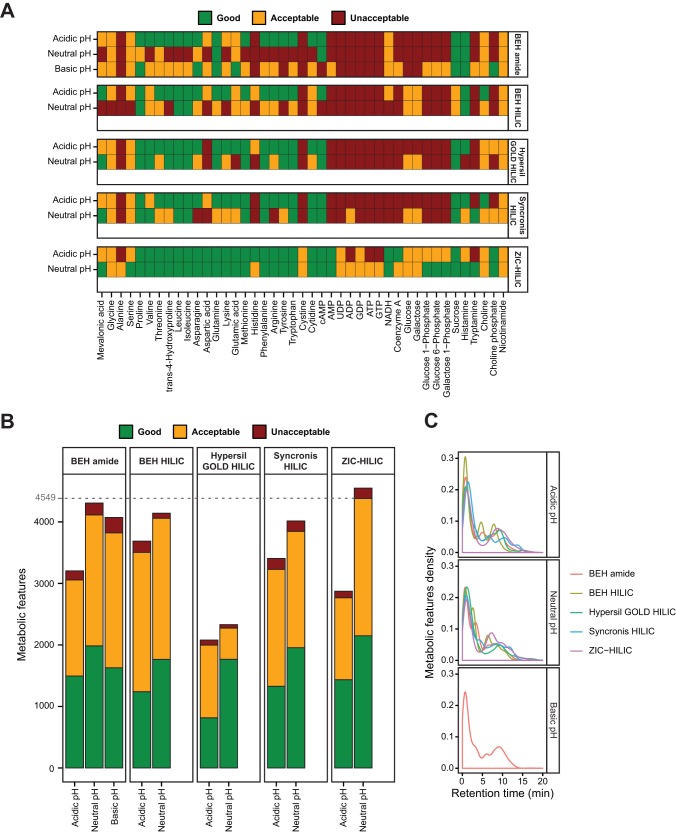
**Optimization of the HILIC-MS analytical procedure.** (*A*) Individual scores of standards under the different HILIC conditions (related to Fig. S1). The best score in positive and negative ESI modes was selected. (*B*) Score of the metabolic features from a urine sample under the different HILIC conditions. Metabolic features from positive and negative ESI modes were combined (related to Fig. S2). (*C*) Repartition of the metabolic features (combination of positive and negative ESI modes) along the chromatographic runs (related to Fig. S2).

To facilitate the comparison of the different chromatographic conditions, we first analyzed a complex mixture of 46 important metabolites with diverse chemical properties (Excel file S1). A qualitative scoring system that assigns a score to each metabolite was created. The score was calculated based on the retention time, peak shape, and MS signal and categorized all metabolites into three groups: “good” (green), “acceptable” (yellow), and “unacceptable” (red). A feature with a good score will: i) retain on the column to avoid ion suppression in the void volume zone; ii) have a narrow elution profile to provide optimal sensitivity and facilitate accurate peak integration; and iii) have an intense MS signal to be accurately extracted, aligned, quantified, and identified. A feature was categorized as acceptable if one of these parameters was not fulfilled. The remaining metabolites were classified as unacceptable, usually because of broad or multiple peaks or low signal intensity. Of the 46 standards, three (*e.g.* adenosine, adenine, and uridine) were excluded from this analysis because they were poorly retained and eluted as broad peaks in all the tested HILIC conditions. Overall, the zwitterionic column ZIC-HILIC operated at neutral pH was superior for separating the 43 tested standards because 100% had a good or acceptable score (Fig. 1*A*). In comparison, 67% had a good or acceptable score with BEH HILIC in acidic conditions. Some representative examples are presented in Fig. S1. Baseline separation of l-leucine and l-isoleucine could only be achieved with the ZIC-HILIC column as was the case for glucose 1-phosphate and glucose 6-phosphate. Similarly, the acidic amino acids (aspartic and glutamic acids) and cystine (oxidation of two cysteine molecules) were only well resolved using the ZIC-HILIC column.

##### Performance of the HILIC Conditions Using Human Urine Samples

We next compared the performance of the same 11 chromatographic conditions in positive and negative ESI modes using urine samples known to contain a great complexity of hydrophilic metabolites. The objective was to maximize the number of detected metabolic features (characterized by a unique mass/charge ratio and retention time) with a good or acceptable score. Among all conditions tested, the ZIC-HILIC column operated at neutral pH provided the best performance with the highest number of metabolic features with a good or acceptable score (4,549) (Fig. 1*B*). This condition was better than BEH amide and BEH HILIC operated at neutral pH, which yielded 4,306 and 4,141 metabolic features with the same scores, respectively. Interestingly, all the tested columns were more efficient at neutral pH than in acidic conditions, and it was more prominent in negative than in positive ESI mode (Fig. S2). The number of metabolic features is known to be a relevant measure of the coverage of a metabolome (28). Importantly, among 9,862 metabolic features extracted with XCMS from a urine sample injected onto a ZIC-HILIC column operated at neutral pH, 2,395 were putatively identified by matching our local database (± 5 ppm). In contrast, BEH amide and BEH HILIC at neutral pH led to the putative identification of 2,163 and 2,235 metabolic features, respectively. Accurate quantification and identification require optimization of the chromatographic separation to minimize ion suppression and mass spectral complexity. Among all the tested columns, fewer features eluted in the void volume zone at neutral pH with ZIC-HILIC (Fig. 1*C* and Fig. S2). Altogether, the ZIC-HILIC column operated at neutral pH provided the optimal results for maximizing the number of hydrophilic metabolites profiled in urine.

Further optimizations were performed by modifying: i) the organic gradient slope using two mobile phases A with different concentration of acetonitrile while running the same program (10 mm ammonium acetate in 5/95 acetonitrile/water *versus* 10 mm ammonium acetate in 50/50 acetonitrile/water), ii) the oven temperature (20 °C *versus* 40 °C), and iii) the flow rate (0.5 ml/min *versus* 0.6 ml/min). The best condition was phase A: 10 mm ammonium acetate in 50/50 acetonitrile/water, oven temperature 40 °C and flow-rate 0.5 ml/min (Fig. S3). Interestingly, very few metabolic profiling studies of urine and plasma were performed using a ZIC-HILIC column, and they all used acidic solvents by adding 0.1% formic acid (39–41). In our experience, using solvents at neutral pH was clearly superior. We also observed that ZIC-HILIC column operated at acidic pH produced better results when 0.2% acetic acid was added to the mobile phases instead of 0.1% formic acid (Fig. S4). Overall, our optimized HILIC-MS procedure provided optimal performances on a large set of hydrophilic standards and enabled the most “comprehensive” analysis of urine samples with the highest number of metabolic features with good or acceptable score as well as the most spread repartition of these features along the chromatographic gradient.

### Suitability of the Optimized HILIC-MS Procedure for the Untargeted Analysis of Hydrophilic Metabolites in Urine and Plasma

**Fig. 2.**
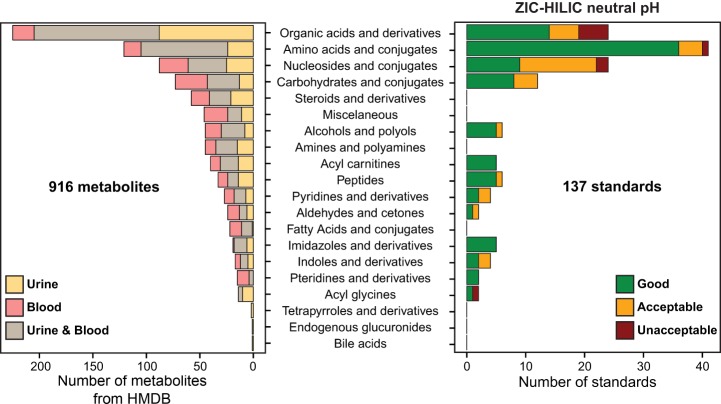
**Suitability of the optimized HILIC-MS procedure for the untargeted analysis of hydrophilic metabolites in urine and plasma.** Number of metabolites labeled as detected and quantified in HMDB in urine, blood, and both classified by chemical classes (*left panel*). The score of 137 standards belonging to the different chemical classes under the optimized HILIC-MS approach is shown on the right panel; 93% of the standards had a good or acceptable score.

In order to gain a preliminary view of the metabolite chemical classes that populate urine and blood, we generated a list of metabolites labeled as detected and quantified in urine, blood, or both in the Human Metabolome Database (HMDB, April 2013). Metabolites with a calculated logP above 3 (likely not retained on the ZIC-HILIC column), inorganic compounds, and metabolites with a mass below 50 Da and above 1,000 Da were excluded from this list. The final list contained 916 unique metabolites among which 272 were found exclusively in urine, 223 in blood, and 421 in both (Excel file S2). It appears that organic acids and derivatives as well as amino acids and conjugates are the two most important chemical classes in urine and blood (Fig. 2).

In order to closely mimic the proportion of metabolites within each chemical classes reported in urine and blood, three other standard mixtures were prepared in addition to the one used above (Excel file S1). Among 174 standards, 37 were excluded from the analysis because they were poorly retained on the ZIC-HILIC column (*i.e.* elution in the void volume zone). These compounds belonged to the following chemical classes: steroids and derivatives, fatty acids and conjugates, most pyridines and derivatives, and most indoles and derivatives. Application of the scoring system showed that the optimized HILIC-MS method performed very well for most of the tested metabolites with 126 out of 137 (93%) exhibiting a good or acceptable score (Fig. 2). Amino acids and conjugates, carbohydrates and conjugates, alcohols and polyols, acyl carnitines, peptides, imidazoles and derivatives, and pteridines and derivatives were particularly well resolved. The compounds with unacceptable scores eluted as broad multiple peaks. Overall, the optimized HILIC procedure is well suited to profile a wide range of hydrophilic metabolites representative of urine and blood compositions. Importantly, many of the compounds that were not well retained or had unacceptable scores were retained and well resolved with the optimized RPLC conditions (see below).

### Optimization of the RPLC-MS Analytical Procedure

Although numerous companies manufacture C18-bonded silica columns, the selectivity of non- and moderately polar compounds can vary greatly depending on the density of C18 alkyl chains, the accessibility of silanol, or the presence of bonding groups between the silica beads and C18 alkyl groups (42). The use of the latter enables a better retention of some polar compounds while conserving RPLC advantages. Among this type of column, BEH C18 and HSS T3 columns are very popular for the global metabolomic profiling of urine (43, 44) and blood (45, 46).

#### 

##### Performance of the RPLC Columns Using Standards

**Fig. 3.**
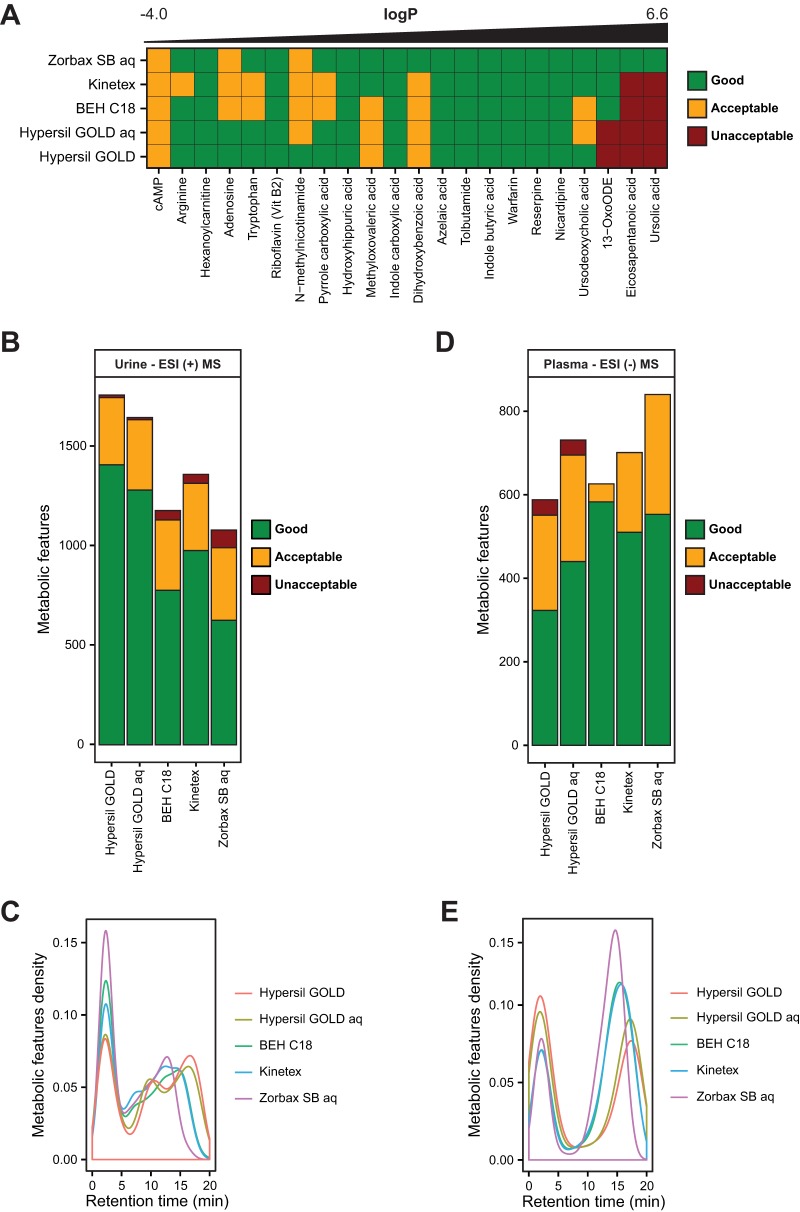
**Optimization of the RPLC-MS analytical procedure.** (*A*) Individual scores of standards with increasing calculated logP values under the different RPLC conditions in negative mode. Score of the metabolic features from (*B*) urine and (*D*) plasma under the different RPLC conditions. Repartition of the metabolic features from (*C*) urine and (*E*) plasma along the chromatographic runs.

The performance of five C18-bonded silica RPLC columns were compared using 22 standards (Excel file S1): Hypersil GOLD, Hypersil GOLD aq, Kinetex, BEH C18, and Zorbax SB aq. The tested compounds had increasing calculated logP values ranging from −4.0 (cAMP) to 6.6 (ursolic acid). Despite similar composition, the different C18 columns had very distinct selectivities. The two most highly hydrophobic standards (eicosapentanoic acid and ursolic acid) were only eluted from the Zorbax SB aq column, indicating that its stationary phase was less hydrophobic than the others (Fig. 3*A*). 13-OxoODE was not eluted from Hypersil GOLD and Hypersil GOLD aq columns, indicating that they were the most hydrophobic columns. In contrast, BEH C18 and Kinetex had intermediary hydrophobicity. When eluted from the column, all the analyzed compounds had a good or acceptable score under all the chromatographic conditions.

##### Performance of the RPLC Columns Using Human Urine and Plasma Samples

We next compared the performance of the RPLC columns for the untargeted metabolomic profiling of urine and plasma samples. The two most hydrophobic columns (Hypersil GOLD, and Hypersil GOLD aq) enabled the analysis of more metabolic features with a good or acceptable score (1,746 and 1,635, respectively) from urine in positive ESI mode (Fig. 3*B*). BEH C18 and Kinetex columns had intermediary performances with 1,131 and 1,360 good or acceptable features whereas Zorbax SB aq yielded the fewest features. Among the two best columns, fewer metabolic features eluted in the void volume zone with Hypersil GOLD than with Hypersil GOLD aq, suggesting that the former was better for optimal peak separation (Fig. 3*C*). Thus, Hypersil GOLD performed the best for the untargeted analysis of urine among the tested columns. This result is likely due to the expectation that most urine metabolites are moderately hydrophobic and thus likely to be better retained on a highly hydrophobic column (12). Despite the popularity of BEH C18 column for the global metabolic profiling of urine (43, 44), the number of putatively identified features was substantially higher with Hypersil GOLD (1,654 *versus* 1,219 in positive mode).

The RPLC columns were also compared for their performance in analyzing plasma samples. Zorbax SB aq enabled the analysis of the highest number of features with a good or acceptable score (840) (Fig. 3*D*). BEH C18 and Kinetex columns yielded 626 and 701 metabolic features with the same scores, respectively. Moreover, a limited amount of features eluted in the void volume zone with Zorbax SB aq (Fig. 3*E*). Altogether, Zorbax SB aq column produced the best results to profile plasma metabolites among the tested RPLC columns. This result might be expected since a large proportion of blood metabolites are very hydrophobic (fatty acids, lipids, and steroids) and will not be eluted from the other more hydrophobic columns (11). Overall, optimal profiling of urine and blood metabolites required different RPLC columns.

### Intra- and Long-Term Interbatch Reproducibility of the Optimized HILIC- and RPLC-MS Procedures

Generating reproducible results is key to successful comprehensive metabolomic profiling studies. In particular, the retention time must be stable for accurate peak alignment and peak selection, and the MS signal must be reproducible for accurate statistical analysis. RPLC-MS is considered more robust than HILIC-MS; hence, the former is often used alone for large-scale untargeted metabolomic studies (43–46), but the metabolome coverage is not optimal. It is known that HILIC requires more equilibration time than RPLC (19, 20). Using 2D principal component analysis plots, we found that that ZIC-HILIC column was adequately equilibrated and conditioned after the injection of 12 biological samples (Fig. S5*A*). Injecting blanks instead of samples did not equilibrate the system because 12 samples were necessary before acquisition of reproducible data (Fig. S5*B*). Injection of five biological matrixes sufficed to equilibrate RPLC-MS systems (data not shown).

#### 

##### Intrabatch Reproducibility

**Fig. 4.**
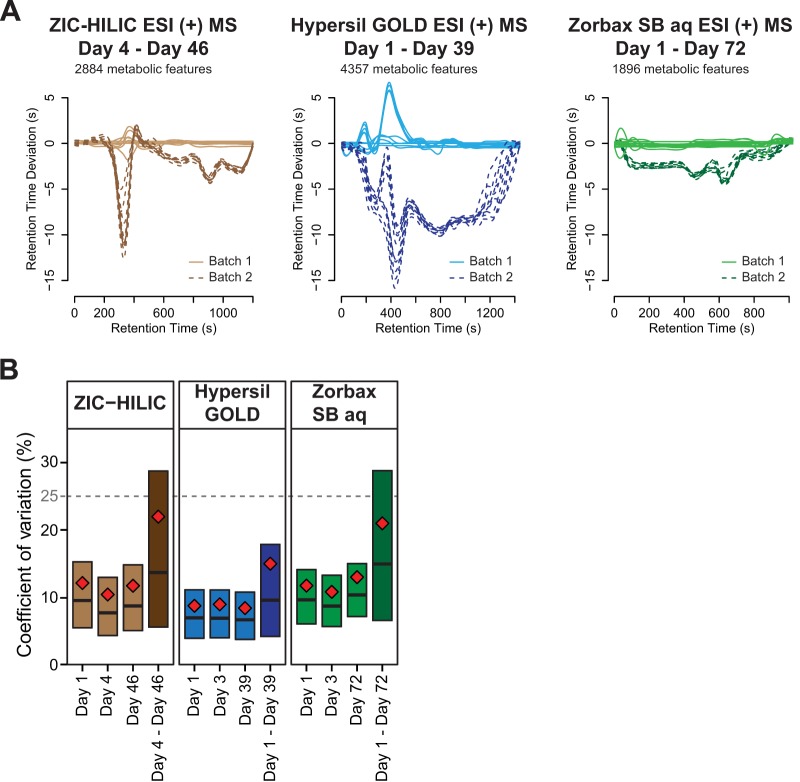
**Intra- and long-term interbatch reproducibility of the optimized HILIC- and RPLC-MS procedures.** (*A*) Intrabatch and long-term interbatch reproducibility of the retention time of metabolic features across two batches analyzed 42, 38 days and 71 days after an initial analysis with ZIC-HILIC, Hypersil GOLD and Zorbax SB aq columns, respectively. Each batch consisted of 10 injections of the same urine sample for ZIC-HILIC and Hypersil GOLD columns and of 10 injections of the same plasma sample for Zorbax SB aq column (related to Fig. S6). (C) Intra- (light color) and long-term interbatch (dark color) reproducibility of peak area (related to Fig. S7). The horizontal black line represents the CV median and the red diamond the CV mean.

To evaluate intrabatch retention time and peak area reproducibility, the same urine (ZIC-HILIC and Hypersil GOLD columns) or plasma sample (Zorbax SB aq) was injected 10 times consecutively after adequate equilibration and conditioning. For HILIC-MS, the retention time deviation was very small with a maximum shift of 2 s for a run that lasts 20 min (< 1% variation) (Fig. 4*A* and Fig. S6*A*). This deviation was consistent between three independent experiments and was very similar to that obtained with the two RPLC methods (*i.e.* Hypersil GOLD and Zorbax SB aq), which had maximum retention time deviations of 7 s and 2 s, respectively (< 1% variation) (Fig. 4*A* and Figs. S6*B* and S6*C*). The intrabatch peak area reproducibility of HILIC-MS was also excellent with an average coefficient of variation (CV) of 11.4% ± 0.9% (Fig. 4*B*) even though the range of metabolite abundances was very wide (order of magnitude ∼5 × 10^4^) (Fig. S7*A*). These values were similar to that obtained with the RPLC methods where the average CVs were 8.7% ± 0.3% and 11.8% ± 1.1% for Hypersil GOLD and Zorbax SB aq columns, respectively (Fig. 4*B*). In summary, after adequate equilibration and conditioning intrabatch reproducibility of the HILIC-MS method was excellent and similar to RPLC-MS procedures.

##### Long-Term Interbatch Reproducibility

Interbatch reproducibility is very important for large-scale untargeted metabolomic studies that involve thousands of samples from longitudinal experiments. For such studies, it is inevitable to process the samples in different batches before combining the data for statistical analysis (47). We therefore assessed the long-term interbatch reproducibility of HILIC- and RPLC-MS (*i.e.* Hypersil GOLD) procedures by injecting the same urine sample 42 and 38 days after an initial analysis, respectively. The columns were moderately used between the two batches with the interim injection of ∼70 samples (standards and biological samples). Retention time deviation was maximum ∼12 s (1% variation) for HILIC-MS and was very similar to that obtained with Hypersil GOLD column (∼16 s corresponding to 1.1% variation) (Fig. 4*A*). Despite a higher retention time variation than that calculated for intrabatch analysis, peak alignment was not impaired. The peak area reproducibility of the optimized HILIC-MS system was good with an average CV of 22.0%, and 70.4% of the metabolic features had a CV ≤ 25%. RPLC-MS (Hypersil GOLD) was more robust with an average CV of 15.0% and 84.7% of the features had a CV ≤ 25% (Fig. 4*B*). We also analyzed MS signal variability of a plasma sample injected onto Zorbax SB aq column 71 days after an initial analysis and found slightly higher variability (average CV of 21.0% and 70.2% of the metabolic features had a CV ≤ 25%) presumably because of independent metabolite extraction, and increased time in storage. Although the long-term interbatch variation of peak area for HILIC-MS was greater than its intrabatch variation, the proportion of reproducible metabolic features was similar to previously reported intrabatch peak areas CVs (28, 48, 49). Altogether, analyzing urine and plasma samples within 40 days with the optimal HILIC- and RPLC-MS procedures enabled acquisition of highly reproducible data.

### Combination of HILIC- and RPLC-MS in Positive and Negative ESI Modes and Expansion of the Metabolome Coverage of Human Urine and Plasma

**Fig. 5.**
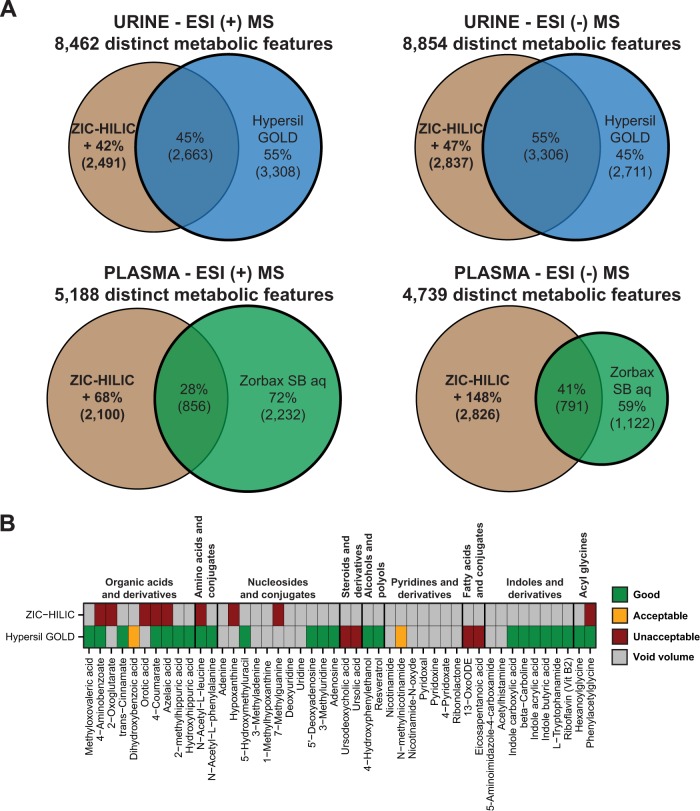
**Combination of HILIC- and RPLC-MS in positive and negative ESI modes and expansion of the metabolome coverage of human urine and plasma.** (*A*) Venn diagrams representing the proportion and quantity of metabolic features detected only in HILIC mode compared with RPLC alone for urine (*top panel*) and plasma (*bottom panel*) samples in positive (*left panel*) and negative (*right panel*) ESI modes (related to Fig. S8). (*B*) Individual scores of the standards that had unacceptable scores or that eluted in the void volume in HILIC mode when injected onto Hypersil GOLD column (related to Fig. S9).

The complementarity between HILIC and RPLC modes was evaluated using the accurate mass (± 10 ppm). 5,971 metabolic features were detected in urine in positive ESI mode by RPLC-MS (Fig. 5*A*). HILIC-MS enabled the detection of 2,491 new distinct metabolic features (+42%) not detected with RPLC-MS. In negative ESI mode, 2,837 new distinct metabolic features were detected with HILIC (+ 47%). Therefore, using HILIC in combination with RPLC expanded the metabolome coverage of urine by ∼44% compared with RPLC alone. For plasma, HILIC enabled the detection of 2,100 (+68%) and 2,826 (+148%) new distinct metabolic features in positive and negative ESI modes, respectively. Hence, HILIC dramatically expanded the metabolome coverage of plasma by ∼108% compared with RPLC alone. Interestingly, very few metabolic features were found in both positive and negative ESI modes, indicating that these modes detect distinct metabolites (Fig. S8*A*) (41, 50). Altogether, combining the optimized HILIC- and RPLC-MS analytical procedures in positive and negative ESI modes enabled the detection of 16,084 and 9,494 distinct metabolic features in urine and plasma, respectively.

Importantly, the overlapping features detected with both HILIC- and RPLC-MS were not necessarily of the same quality in both modes. For example, a metabolite that was eluted in the void volume zone with RPLC and retained with HILIC will be of higher quality in the latter because its quantification will be more robust and its identification easier. Some examples including sn-glycero-phosphocholine, carnosine, and l-ornithine are presented Fig. S9*A*. Interestingly, 70% of the overlapping metabolic features in urine were retained in HILIC mode (outside of the void volume zone), indicating that these metabolites will be of better quality in this mode. Moreover, 50% of the 46 standards eluting in the void volume or with an unacceptable score in HILIC mode had a good or acceptable score with RPLC (Fig. 5*B*). Some examples including N-acetyl-l-leucine, phenylacetylglycine, and indole-2-carboxylic acid are presented Fig. S9*B*. In particular, organic acids and derivatives, amino acids and conjugates, alcohols and polyols, indoles and derivatives, and acyl glycines were well resolved with RPLC. However, nucleosides and conjugates as well as pyridines and derivatives eluted in the void volume with both systems. As already shown above, steroids and derivatives as well as fatty acids and conjugates that were not eluted from Hypersil GOLD column were well resolved with Zorbax SB aq (Fig. 3*A*). Altogether, 88% of the 174 analyzed standards had a good or acceptable score when ZIC-HILIC and Hypersil GOLD columns are used in combination (Fig. S8*B*).

## DISCUSSION

Most untargeted metabolomic studies of urine and plasma are performed by RPLC-MS alone and often with suboptimal conditions. This observation motivated us to find an optimal chromatographic solution involving orthogonal HILIC and RPLC coupled to mass spectrometry that will enable the optimal reproducible coverage of human urine and plasma metabolites. First, we systematically investigated the performance of five HILIC columns with different chemistry operated at three different pH using model compounds and biological samples. We have focused our efforts on varying the nature of the stationary phase and the mobile phase pH because several studies describing the development of HILIC methods have indicated that they are the two most critical parameters for tuning metabolite retention, peak shape, and MS signal intensity (23, 24, 37). Indeed, these two parameters will dictate the amount and type of forces involved in the retention mechanism of the metabolites. In the context of global metabolic profiling, they will impact the quantity and the quality of detected metabolic features. The zwitterionic column ZIC-HILIC operated at neutral pH was superior for separating urine and plasma metabolites with the best peak shape in many cases. Even though the gradient condition, the oven temperature, and the flow rate have been shown to be secondary parameters (37), they were also optimized in HILIC mode. Additionally, we explored the performance of five C18-bonded silica RPLC columns using standards of varying hydrophobicity and biological samples. Hypersil GOLD and Zorbax SB aq columns gave the optimal results for urine and plasma, respectively.

Importantly, after adequate equilibration and conditioning the optimized HILIC method showed excellent intrabatch reproducibility of both retention time and peak area that was similar to those of RPLC-MS procedures. Also, even though more variable, HILIC- and RPLC-MS procedures exhibited similarly good long-term (40 days) interbatch reproducibility. The difference between intra- and long-term interbatch robustness of HILIC-MS was presumably not due to sample degradation during storage because HILIC- and RPLC-MS analyses were performed with the same samples and RPLC-MS interbatch variability was lower. The number of features extracted from the two batches with HILIC-MS was significantly lower on the second batch (3,331 on day 4 compared with 2,325 features on day 46) (Fig. S6*A*). This difference was due to the degradation of the condition of the HILIC column presumably because of the repeated injection of salts contained in urine samples. This degradation caused peak broadening and impacted sensitivity since some low abundance features dropped below the intensity threshold. Despite a clear loss of sensitivity, intrabatch peak area reproducibility on day 46 was as good as on day 4 (Fig. 4*B*). The repeated injection of salts on RPLC column did not impact its condition because unlike HILIC columns, salts are not retained on RPLC columns. Hence, the Hypersil GOLD column performed similarly between the two batches; 4,150 aligned features on day 1 compared with 4,174 on day 39 with similar retention time and peak area reproducibility (Fig. 4*B* and Fig. S6*B*). A significant concern for large-scale metabolomic studies is the interbatch normalization of the data. Indeed, the interbatch variability of untargeted analyses cannot be easily corrected by using a small number of internal standards because the signal drift is nonlinear and depends on each metabolite (51). Some data-driven normalization methods have shown to be efficient (15, 51), and in our case, the cubic spline normalization enabled to obtain the lowest interbatch peak area CV (Figs. S7*B* and 7*C*).

Finally, the complementarity of the optimized HILIC- and RPLC-MS analytical procedures in positive and negative ESI modes was evaluated for urine and plasma samples. Combining the optimized HILIC- and RPLC-MS was found to greatly expand the metabolome coverage compared with RPLC alone with 44% and 108% new metabolic features detected enabling the monitoring of over 16,000 and 9,000 distinct features in urine and plasma, respectively. We, therefore, believe that using these optimized analytical procedures will enable more comprehensive untargeted metabolic studies of human body fluids and thus will be of great value for monitoring and discovering metabolic markers of health and disease and for providing insights into human physiology.

## Supplementary Material

Supplemental Data

## Footnotes

^1^ The abbreviations used are:

HILIC: hydrophilic interaction liquid chromatography
RPLC: reverse-phase liquid chromatography
HMDB: human metabolome database.
