# Supplementary material for: Optimized Analytical Procedures for the Untargeted Metabolomic Profiling of Human Urine and Plasma by Combining Hydrophilic Interaction (HILIC) and Reverse-Phase Liquid Chromatography (RPLC)–Mass Spectrometry
Source: Mol Cell Proteomics. 2015 Mar 18;14(6):1684–95. doi: 10.1074/mcp.M114.046508 (PMC4458729; doi:10.1074/mcp.M114.046508)
Supplement: Supplemental Data [file supp_14_6_1684__index.html]

Optimized Analytical Procedures for the Untargeted Metabolomic Profiling of Human Urine and Plasma by Combining Hydrophilic Interaction and Reverse-Phase Liquid Chromatography - Mass Spectrometry — Optimized Analytical Procedures for the Untargeted Metabolomic Profiling of Human Urine and Plasma by Combining Hydrophilic Interaction (HILIC) and Reverse-Phase Liquid Chromatography (RPLC)–Mass Spectrometry — Analytical Platform for Urine and Plasma Metabolomics — Supplemental Data 

# Optimized Analytical Procedures for the Untargeted Metabolomic Profiling of Human Urine and Plasma by Combining Hydrophilic Interaction (HILIC) and Reverse-Phase Liquid Chromatography (RPLC)–Mass Spectrometry

## Supplemental Data

**Files in this Data Supplement:**

- Supplementary experimental section and figures - Supplementary experimental section and figures
- Excel file S1 - Excel file S1
- Excel file S2 - Excel file S2
