# Supplementary material for: Optimized Analytical Procedures for the Untargeted Metabolomic Profiling of Human Urine and Plasma by Combining Hydrophilic Interaction (HILIC) and Reverse-Phase Liquid Chromatography (RPLC)–Mass Spectrometry
Source: Mol Cell Proteomics. 2015 Mar 18;14(6):1684–95. doi: 10.1074/mcp.M114.046508 (PMC4458729; doi:10.1074/mcp.M114.046508)
Supplement: Supplemental Data [file supp_M114.046508_mcp.M114.046508-1.pdf]

Supporting Information

Optimized Analytical Procedures for the Untargeted Metabolomic Profiling of Human Urine and Plasma by Combining Hydrophilic Interaction and Reverse-Phase Liquid Chromatography – Mass Spectrometry

Kévin Contrepois, Lihua Jiang, and Michael Snyder*

Department of Genetics, Stanford University School of Medicine, Stanford, CA, USA.

Corresponding Author

*To whom correspondence should be addressed. E-mail: mpsnyder@stanford.edu

Table of contents

- **Supplementary experimental section**
- **Supplementary figures (Figures S1 to S9)**
- **Supplementary files**

Excel File S1. List of the analytical-grade standards used in the study with their reference number and manufacturer name (HILIC and RPLC).

Excel File S2. List of metabolites labeled as detected and quantified in urine, blood or both in the Human Metabolome Database (HMDB, April 2013) categorized by chemical classes.

SUPPLEMENTARY EXPERIMENTAL SECTION

HILIC and RPLC Column Specifications.

Chromatographic mode	Column name (supplier)	Column type (abbreviation)	Dimensions, particle and pore sizes	pH stability of column
HILIC	BEH amide (Waters)	Ethylene Bridged Hybrid silica + carbamoyl (BEH amide)	2.1 x 50 mm, 1.7 µm, 130Å	2 - 11
	BEH HILIC (Waters)	Ethylene Bridged Hybrid silica (BEH SiO_2)	2.1 x 50 mm, 1.7 µm, 130Å	1 - 9
	Hypersil GOLD HILIC (Thermo Scientific)	Polyethyleneimine (PEI)	2.1 x 50 mm, 1.9 µm, 175Å	2 - 8
	Syncronis HILIC (Thermo Scientific)	Sulfobetaine	2.1 x 100 mm, 1.7 µm, 100Å	2 - 8
	ZIC-HILIC (Merck Millipore)	Sulfobetaine	2.1 x 100 mm, 3.5 µm, 200Å	3 - 8
RPLC	Hypersil GOLD (Thermo Scientific)	C18	2.1 x 150 mm, 1.9 µm, 175Å	1 – 11
	Hypersil GOLD aq (Thermo Scientific)	C18 with embedded polar group	2.1 x 150 mm, 1.9 µm, 175Å	2 - 9
	BEH C18 (Waters)	Ethylene Bridged Hybrid silica + C18	2.1 x 100 mm, 1.7 µm, 130Å	1 - 12
	Kinetex (Phenomenex)	C18	2.1 x 100 mm, 2.6 µm, 100Å	1.5 – 10
	Zorbax SB aq (Agilent Technologies)	C18 with embedded polar group	2.1 x 50 mm, 1.8 µm, 80Å	1 - 8

RPLC Chromatographic Conditions

Column name	Gradient	Flow rate
Hypersil GOLD	1% to 80% B in 10 min	0.25 ml/min
Hypersil GOLD aq	1% to 80% B in 10 min	0.25 ml/min
BEH C18	1% to 80% B in 10 min	0.4 ml/min
Kinetex	1% to 80% B in 10 min	0.4 ml/min
Zorbax SB aq	1% to 80% B in 9 min	0.6 ml/min

Scoring System. Retention time, peak shape and peak area were obtained with MassHunter Qualitative Analysis Software B.05.00 (Agilent Technologies). The score of each standard and metabolic feature (biological sample) was determined using the scoring system as follow:

$$\mathbf{Score_{total} = Score_{retention\ time} + Score_{peak\ shape} + Score_{sensitivity}}$$

HILIC & RPLC	Parameters for standards	Parameters for metabolic features	Score
$\mathbf{Score_{retention\ time}}$[a]	$RT < ½ RT_{d=0.05}$	$RT < ½ RT_{d=0.05}$	-1
	$½ RT_{d=0.05} \leq RT < RT_{d=0.05}$	$½ RT_{d=0.05} \leq RT < RT_{d=0.05}$	0
	$RT \geq RT_{d=0.05}$	$RT \geq RT_{d=0.05}$	1
$\mathbf{Score_{peak\ shape}}$	Width < 0.5 min	Width < 0.5 min	1

	0.5 min ≤ Width < 1.0 min	0.5 min ≤ Width < 1.0 min	0
	1.0 min ≤ Width < 2.5 min	1.0 min ≤ Width < 2.5 min	-1
	Width ≥ 2.5 min	Width ≥ 2.5 min	-3
	Multiple peaks	N/A[b]	-3
**Score$_{sensitivity}$**	Area ≥ 200,000	Area ≥ 200,000	1
	50,000 ≤ Area < 200,000	50,000 ≤ Area < 200,000	0
	10,000 ≤ Area < 50,000	10,000 ≤ Area < 50,000	-1
	Area < 10,000	Area < 10,000	-3

[a] For each chromatographic condition, the void volume zone has been delimitated by calculating the retention time corresponding to a density of metabolic feature of 0.05 using a urine sample.
[b] Not Applicable.

If the Score$_{total}$ of a metabolic feature was above or equal 2, it was categorized as "Good". If the Score$_{total}$ was below 2 and above or equal 0, the score was "Acceptable". If the Score$_{total}$ was below 0 the feature was "Unacceptable".

Parameters used with XCMS and CAMERA. When the score was not calculated, the raw LC-MS files were first converted to mzData.xml files with MassHunter Qualitative Analysis Software and then processed with the XCMS package (version 1.39.4) in R (version 3.0.1). XCMS was used for peak extraction, alignment and quantification with the following parameters:

Category	Sub-category	Parameters ZIC-HILIC	Parameters Hypersil GOLD	Parameters Zorbax SB aq
Feature detection	Method	centWave	centWave	centWave
	ppm	10	10	10
	Minimum peak width	10	10	5
	Maximum peak width	60	45	30
	Signal/Noise threshold	6	6	6
	mzdiff	0.01	0.01	0.01
	Integration method	2	2	2
Retention time	Method	Obiwarp	Obiwarp	Obiwarp
	profStep	1	1	1
Alignment	mzwid	0.015	0.015	0.015
	minfrac	0.75	0.75	0.75
	bw	10	5	4
	max	100	100	100
	minsamp	1	1	1

CAMERA package (version 1.16.0) was used in R to annotate the metabolic features (isotopes, adducts, in-source fragments) before putative identification. The parameters used with CAMERA were as follow:

Category	Sub-category	Parameters
groupFWHM	sigma	6
	perfwhm	0.6
findIsotopes	maxcharge	1
	maxiso	3
	minfrac	0.75
	ppm	10

	mzabs	0.015
groupCorr	cor_eic_th	0.75
	graphMethod	hcs
	pval	0.05
findAdducts	ppm	10
	mzabs	0.015
	multiplier	2

SUPPLEMENTARY FIGURE LEGENDS

Figure S1. Extracted ion chromatograms of selected standards under the different HILIC conditions. Acidic pH = pH 3.4, Neutral pH = pH 6.9, Basic pH = pH 10.15 (related to **Fig. 1**).

Figure S2. Optimization of the HILIC-MS analytical procedure in positive and negative ESI modes. Score of the metabolic features from a urine sample under the different HILIC conditions and their repartition along the chromatographic runs in (A - B) positive and (C - D) negative ESI mode (related to **Fig. 1**).

Figure S3. Optimization of the concentration of organic solvent in mobile phase A, oven temperature, and flow rate in HILIC mode. (A) Score of the metabolic features from a urine sample under the different HILIC conditions. Metabolic features from positive and negative ESI modes were combined. (B) Repartition of the metabolic features (combination of positive and negative ESI modes) along the chromatographic runs.

Figure S4. Performance of HILIC columns operated at acidic pH by adding 0.1% formic acid or 0.2% acetic acid in the mobile phases. (A) Individual scores of standards under the different HILIC conditions. The best score in positive and negative ESI modes was selected. (B) Score of the metabolic features extracted from a urine sample under the different HILIC conditions. Metabolic features from positive and negative ESI modes were combined. (C) Repartition of the metabolic features (combination of positive and negative ESI modes) along the chromatographic runs.

Figure S5. Optimal equilibration and conditioning condition of the HILIC-MS system. (A) 2D PCA plot showing the variation of 24 injections of the same urine sample onto the ZIC-HILIC column at neutral pH. After 12 injections (red dots), the signal is more reproducible (green dots). (B) 2D PCA plot showing the variation of 24 injections of the same urine sample after the injection of 12 blanks onto the ZIC-HILIC column at neutral pH. The injection of 12 blanks did not increase the signal stability of the first 12 injections (red dots). 2D-PCA plots were generated using MetaboAnalyst 2.0 website (www.metaboanalyst.ca) after interquartile range (IQR) normalization, logarithmic transformation and autoscaling of the data.

Figure S6. Intra-batch reproducibility of the retention time. Three independent batches are shown that consist of 10 consecutive injections of the same urine sample onto (A) ZIC-HILIC and (B) Hypersil GOLD columns or 10 consecutive injections of the same plasma sample onto (C) Zorbax SB aq column (related to **Fig. 4**).

Figure S7. Intra- and long-term inter-batch normalization. (A) Reproducibility of the MS signal between two consecutive injections of the same sample according to their MS signal intensity with the three optimized LC-MS systems. The order of magnitude between the most and least abundant features is shown. Effect of data-driven normalization on (B) intra-batch and (C) long-term inter-batch reproducibility of peak area (related to **Fig. 4**). NO = Before normalization, LBN = linear baseline normalization, PQN = probabilistic quotient normalization, CLN = cyclic LOESS normalization, CSN = cubic spline normalization, QN = quantile normalization. Normalization of the data was performed as previously described (51). Cubic spline normalization gave the lowest intra- and inter-batch CVs.

Figure S8. Complementarity of positive and negative ESI modes for the optimized HILIC- and RPLC-MS analytical procedures. (A) Venn diagrams representing the proportion and quantity of overlapping and distinct metabolic features measured in positive and negative ESI modes (related to **Fig. 5**). (B) Complementarity of ZIC-HILIC and Hypersil GOLD columns toward 174 standards classified by chemical classes. The third row at the bottom shows that 88% of the standards have a “Good” or “Acceptable” score when ZIC-HILIC and Hypersil GOLD columns are combined.

Figure S9. Extracted ion chromatograms of selected standards using the optimized HILIC- and RPLC-MS procedures. (A) Example of compounds that are well retained on the HILIC column and eluted in the void volume with RPLC. (B) Example of compounds that elute as very wide multiple peaks on the HILIC column but have a good chromatographic behavior with RPLC.

Figure S1.

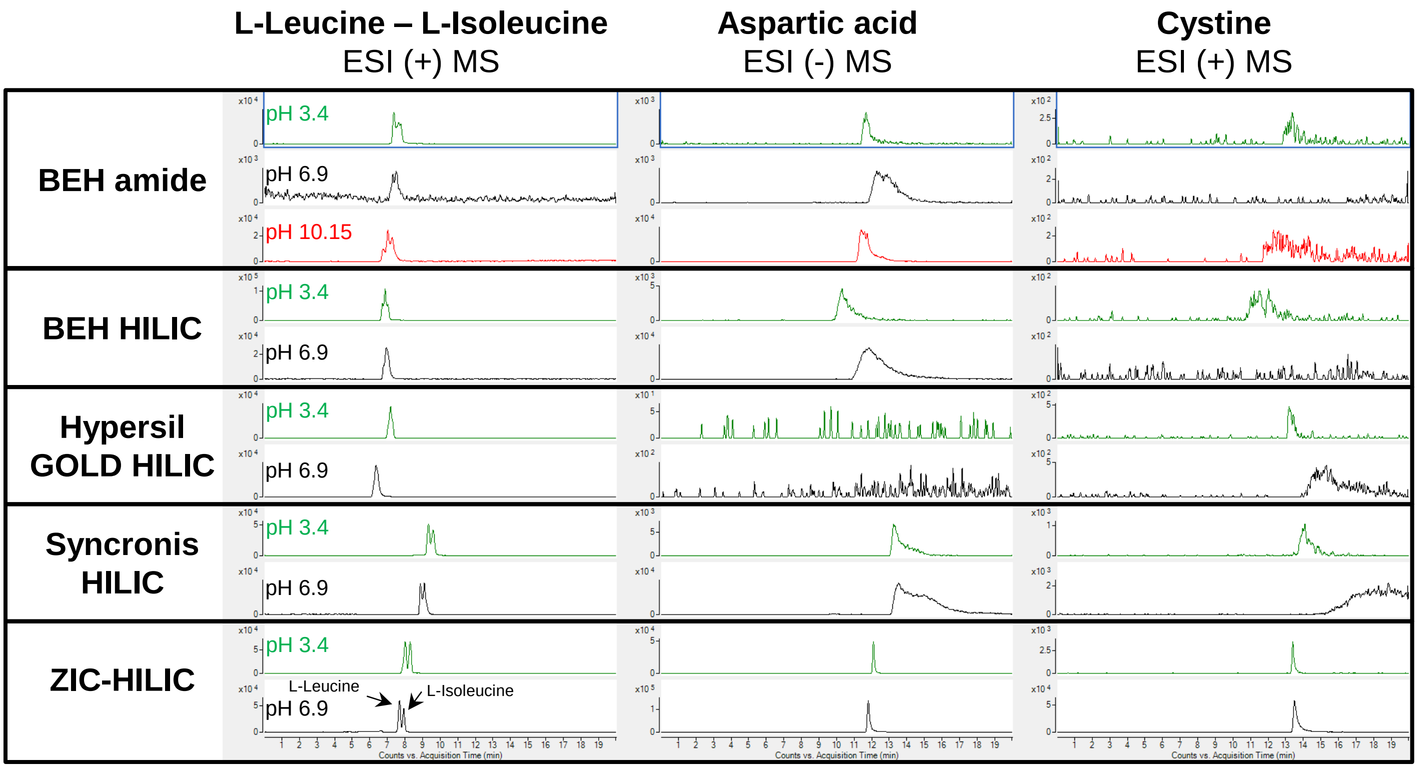
L-Leucine – L-Isoleucine
ESI (+) MS
Aspartic acid
ESI (-) MS
Cystine
ESI (+) MS
BEH amide
BEH HILIC
Hypersil GOLD HILIC
Syncronis HILIC
ZIC-HILIC
pH 3.4
pH 6.9
pH 10.15
L-Leucine
L-Isoleucine
Counts vs. Acquisition Time (min)

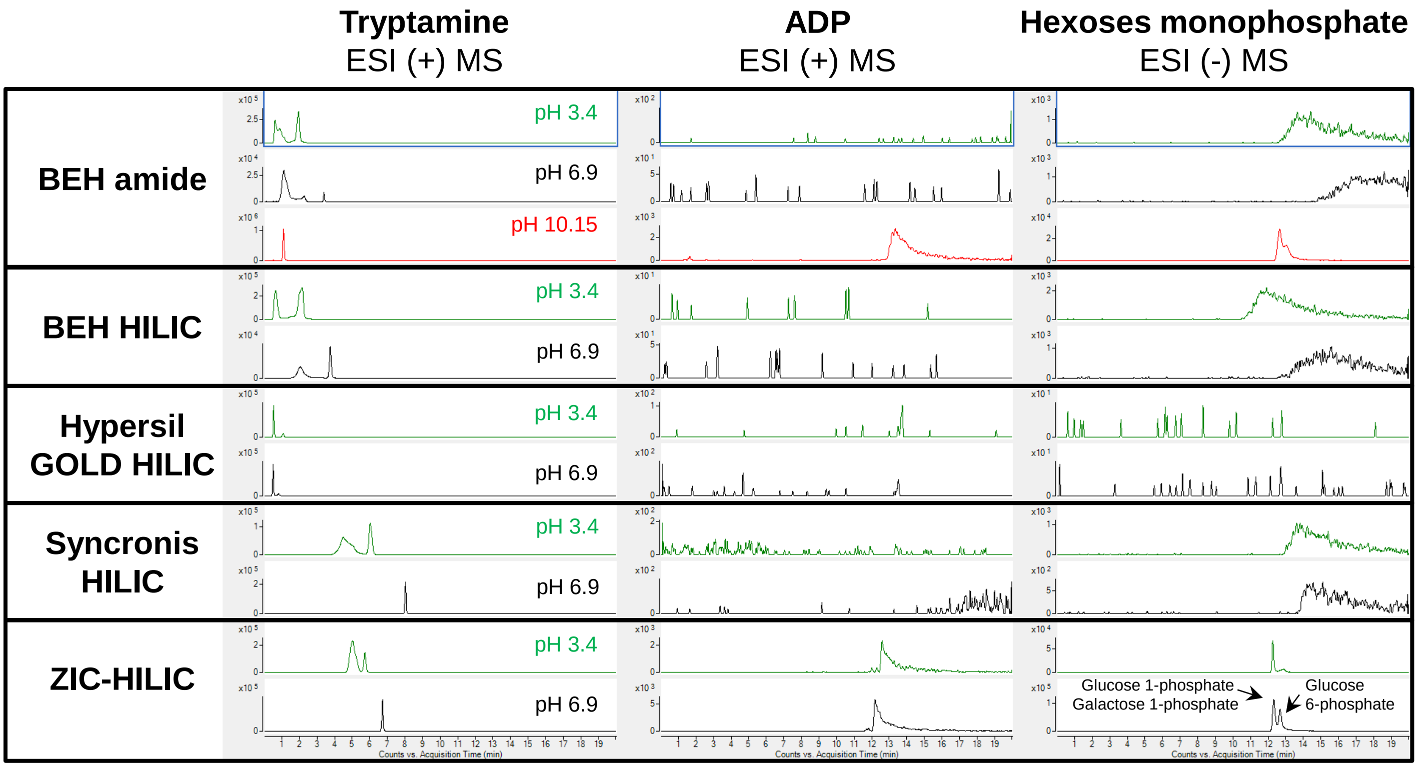
Tryptamine
ESI (+) MS
ADP
ESI (+) MS
Hexoses monophosphate
ESI (-) MS
BEH amide
BEH HILIC
Hypersil GOLD HILIC
Syncronis HILIC
ZIC-HILIC
pH 3.4
pH 6.9
pH 10.15
Glucose 1-phosphate
Galactose 1-phosphate
Glucose 6-phosphate
Counts vs. Acquisition Time (min)

Figure S2.

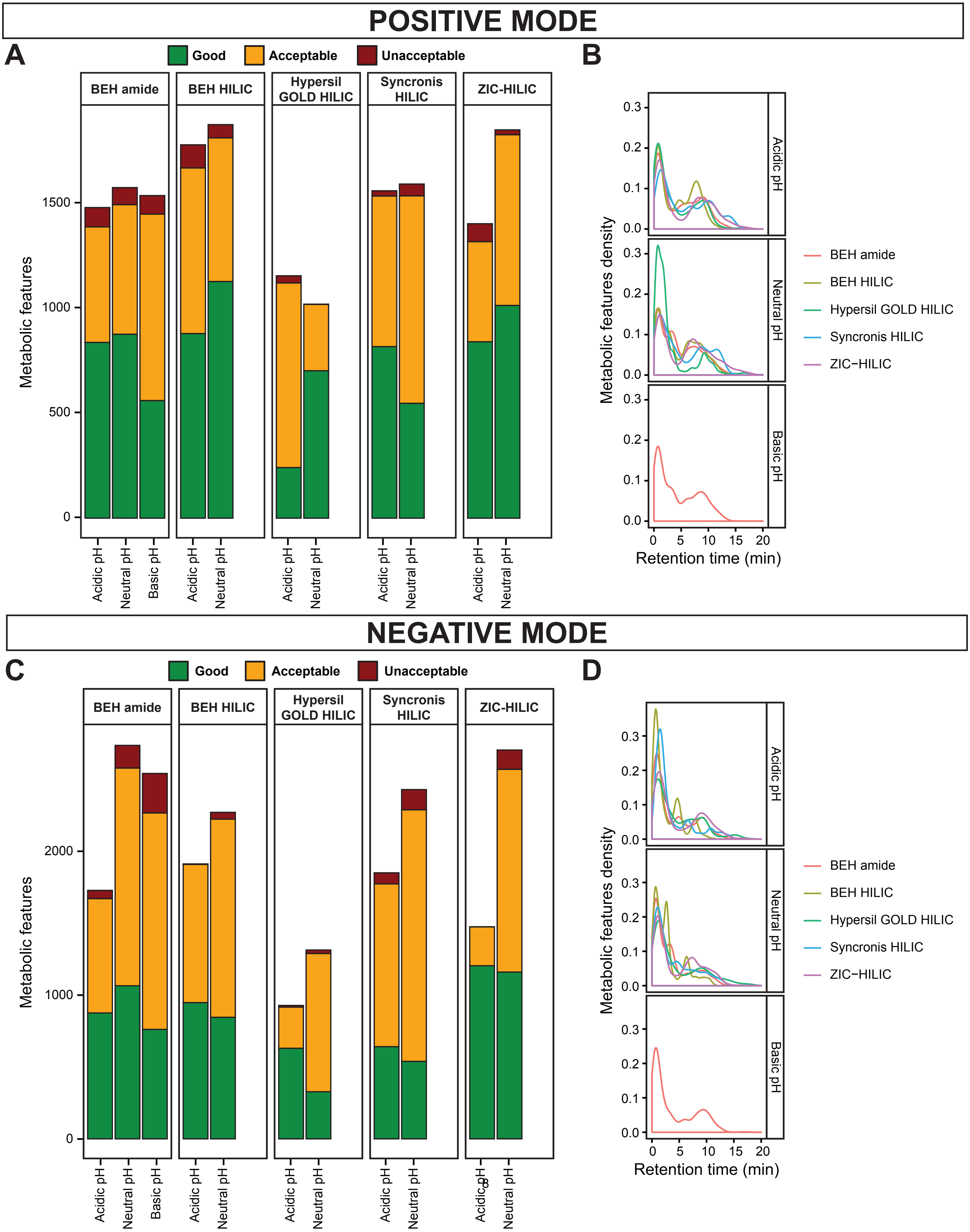
POSITIVE MODE
A
Good
Acceptable
Unacceptable
BEH amide
BEH HILIC
Hypersil GOLD HILIC
Syncronis HILIC
ZIC-HILIC
Metabolic features
0
500
1000
1500
Acidic pH
Neutral pH
Basic pH
B
Metabolic features density
0.0
0.1
0.2
0.3
Acidic pH
Neutral pH
Basic pH
0
5
10
15
20
Retention time (min)
BEH amide
BEH HILIC
Hypersil GOLD HILIC
Syncronis HILIC
ZIC−HILIC
NEGATIVE MODE
C
Good
Acceptable
Unacceptable
BEH amide
BEH HILIC
Hypersil GOLD HILIC
Syncronis HILIC
ZIC-HILIC
Metabolic features
0
1000
2000
Acidic pH
Neutral pH
Basic pH
D
Metabolic features density
0.0
0.1
0.2
0.3
Acidic pH
Neutral pH
Basic pH
0
5
10
15
20
Retention time (min)
BEH amide
BEH HILIC
Hypersil GOLD HILIC
Syncronis HILIC
ZIC−HILIC

Figure S3.

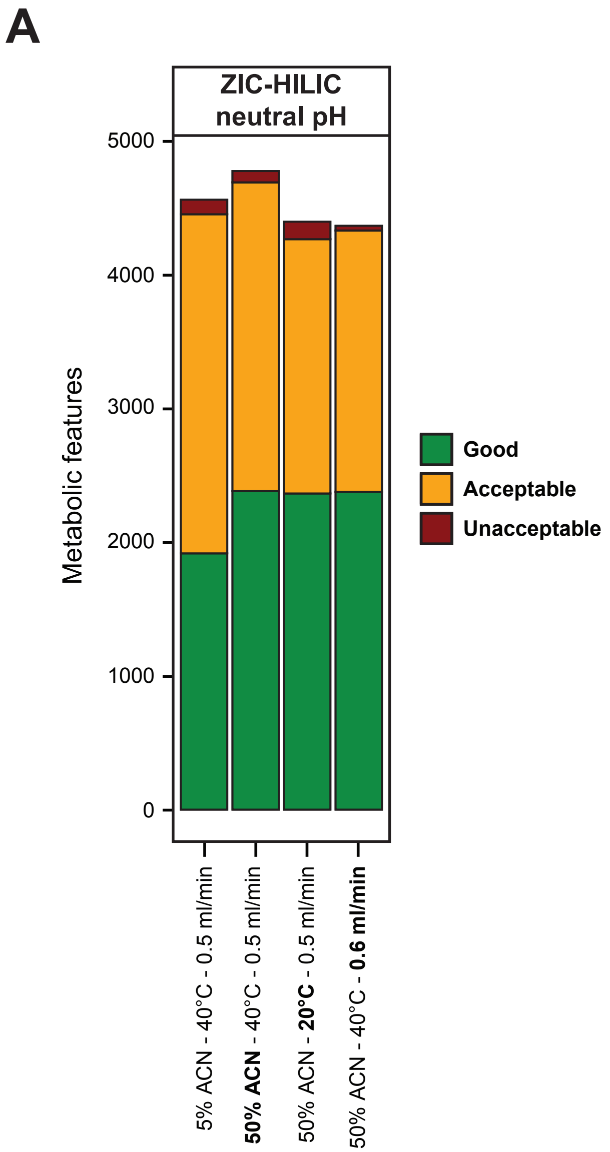
A
ZIC-HILIC
neutral pH
Metabolic features
5000
4000
3000
2000
1000
0
Good
Acceptable
Unacceptable
5% ACN - 40°C - 0.5 ml/min
50% ACN - 40°C - 0.5 ml/min
50% ACN - 20°C - 0.5 ml/min
50% ACN - 40°C - 0.6 ml/min

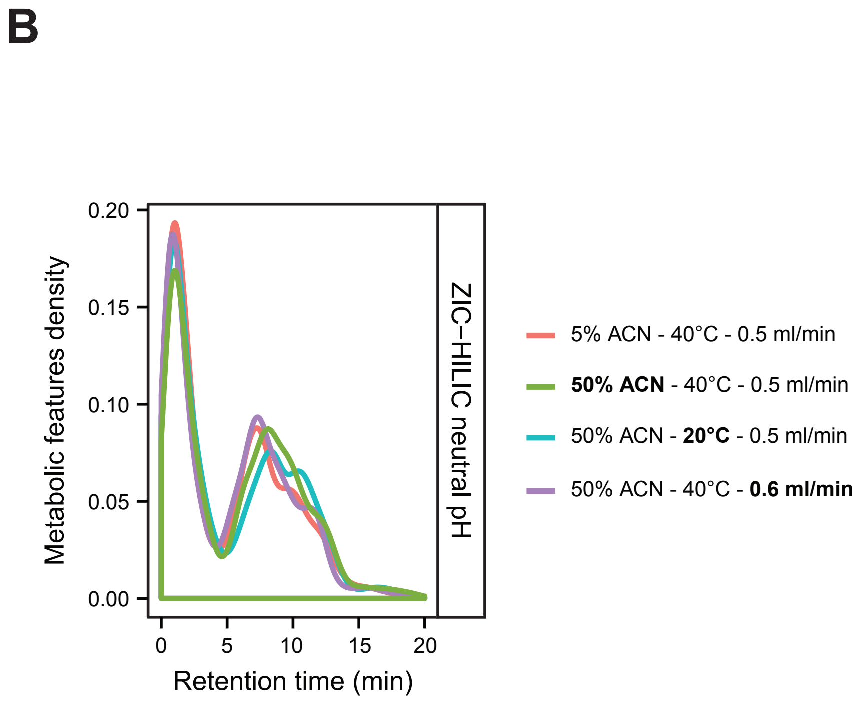
B
Metabolic features density
0.20
0.15
0.10
0.05
0.00
0
5
10
15
20
Retention time (min)
ZIC−HILIC neutral pH
5% ACN - 40°C - 0.5 ml/min
50% ACN - 40°C - 0.5 ml/min
50% ACN - 20°C - 0.5 ml/min
50% ACN - 40°C - 0.6 ml/min

Figure S4.

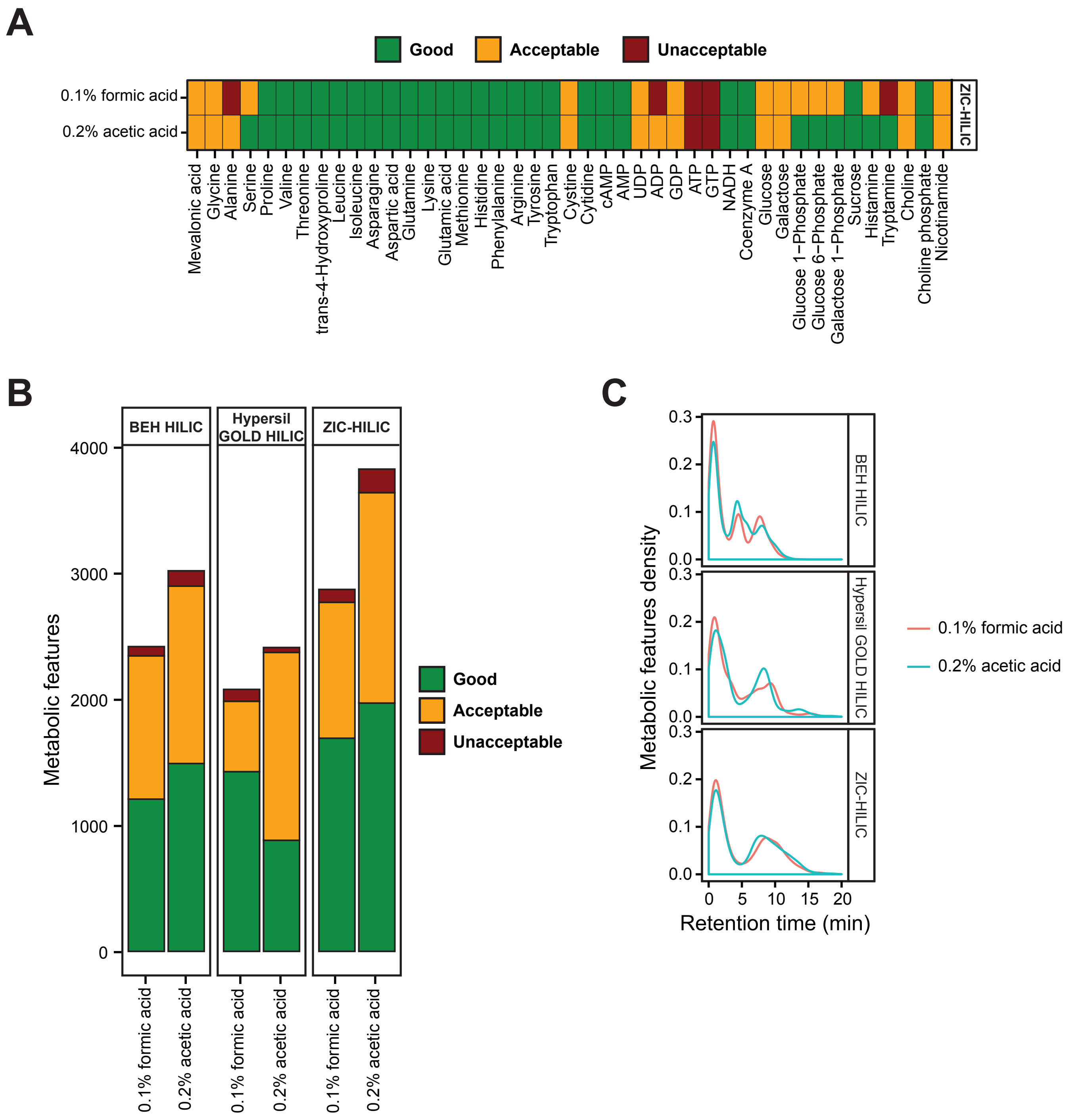
A
Good
Acceptable
Unacceptable
0.1% formic acid
0.2% acetic acid
ZIC-HILIC
Mevalonic acid
Glycine
Alanine
Serine
Proline
Valine
Threonine
trans-4-Hydroxyproline
Leucine
Isoleucine
Asparagine
Aspartic acid
Glutamine
Lysine
Glutamic acid
Methionine
Histidine
Phenylalanine
Arginine
Tyrosine
Tryptophan
Cystine
Cytidine
cAMP
AMP
UDP
ADP
GDP
ATP
GTP
NADH
Coenzyme A
Glucose
Galactose
Glucose 1−Phosphate
Glucose 6−Phosphate
Galactose 1−Phosphate
Sucrose
Histamine
Tryptamine
Choline
Choline phosphate
Nicotinamide
B
BEH HILIC
Hypersil GOLD HILIC
ZIC-HILIC
Metabolic features
4000
3000
2000
1000
0
0.1% formic acid
0.2% acetic acid
Good
Acceptable
Unacceptable
C
Metabolic features density
0.3
0.2
0.1
0.0
BEH HILIC
Hypersil GOLD HILIC
ZIC-HILIC
0
5
10
15
20
Retention time (min)
0.1% formic acid
0.2% acetic acid

Figure S5.

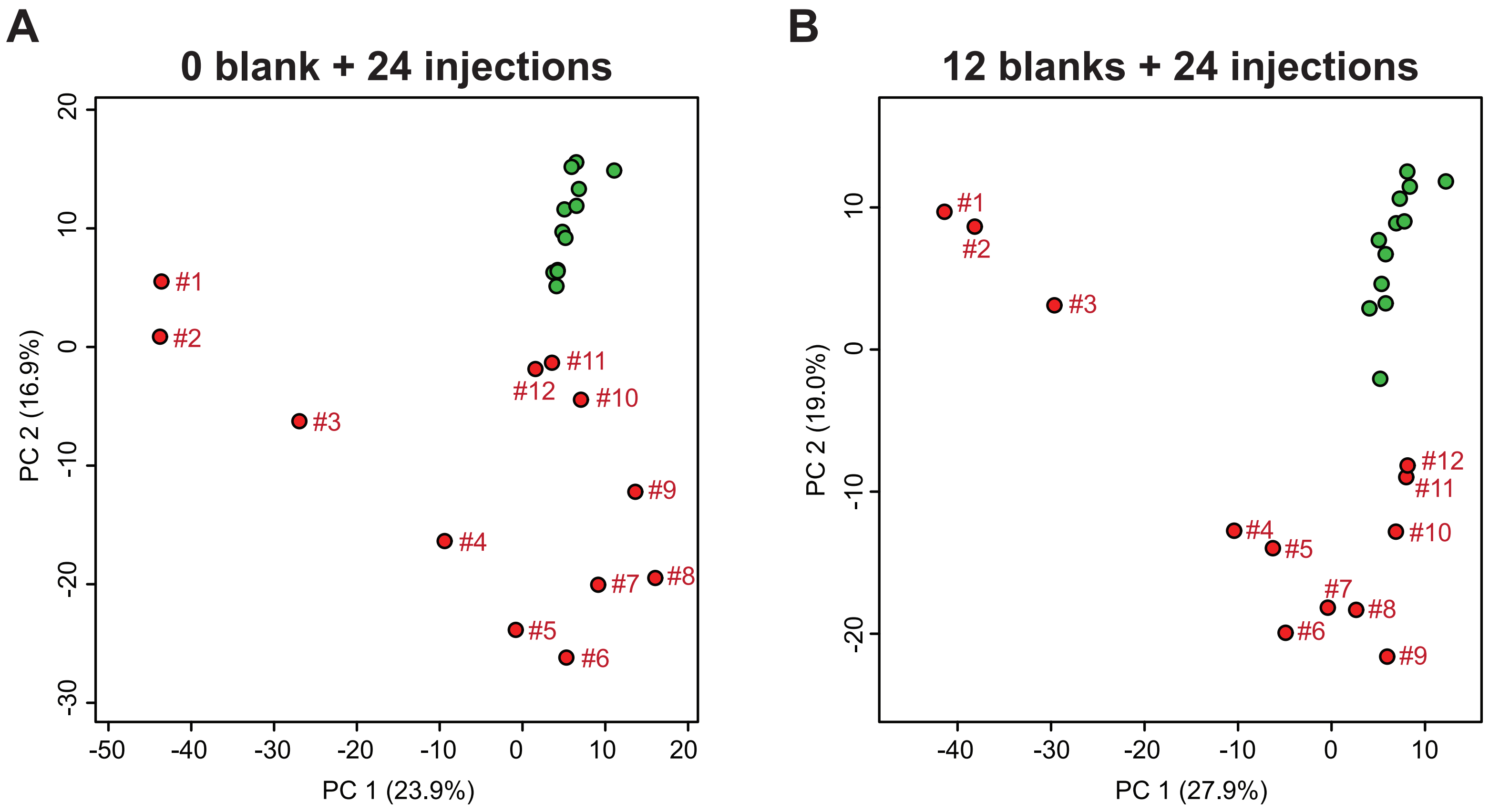
A
0 blank + 24 injections
PC 2 (16.9%)
PC 1 (23.9%)
#1
#2
#3
#4
#5
#6
#7
#8
#9
#10
#11
#12
B
12 blanks + 24 injections
PC 2 (19.0%)
PC 1 (27.9%)
#1
#2
#3
#4
#5
#6
#7
#8
#9
#10
#11
#12

Figure S6.

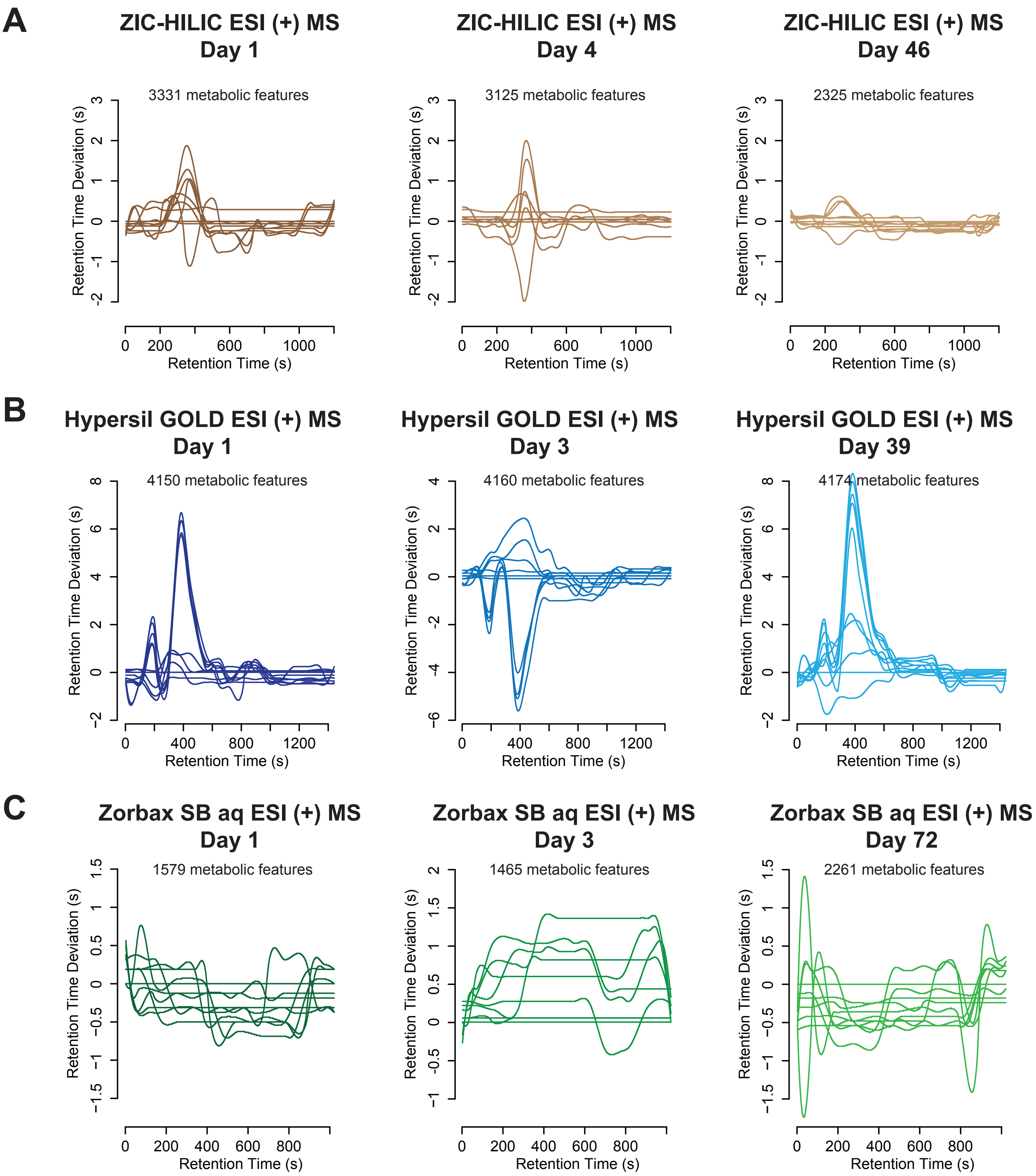

A
ZIC-HILIC ESI (+) MS
Day 1
3331 metabolic features
ZIC-HILIC ESI (+) MS
Day 4
3125 metabolic features
ZIC-HILIC ESI (+) MS
Day 46
2325 metabolic features
B
Hypersil GOLD ESI (+) MS
Day 1
4150 metabolic features
Hypersil GOLD ESI (+) MS
Day 3
4160 metabolic features
Hypersil GOLD ESI (+) MS
Day 39
4174 metabolic features
C
Zorbax SB aq ESI (+) MS
Day 1
1579 metabolic features
Zorbax SB aq ESI (+) MS
Day 3
1465 metabolic features
Zorbax SB aq ESI (+) MS
Day 72
2261 metabolic features
Retention Time Deviation (s)
Retention Time (s)

Figure S7.

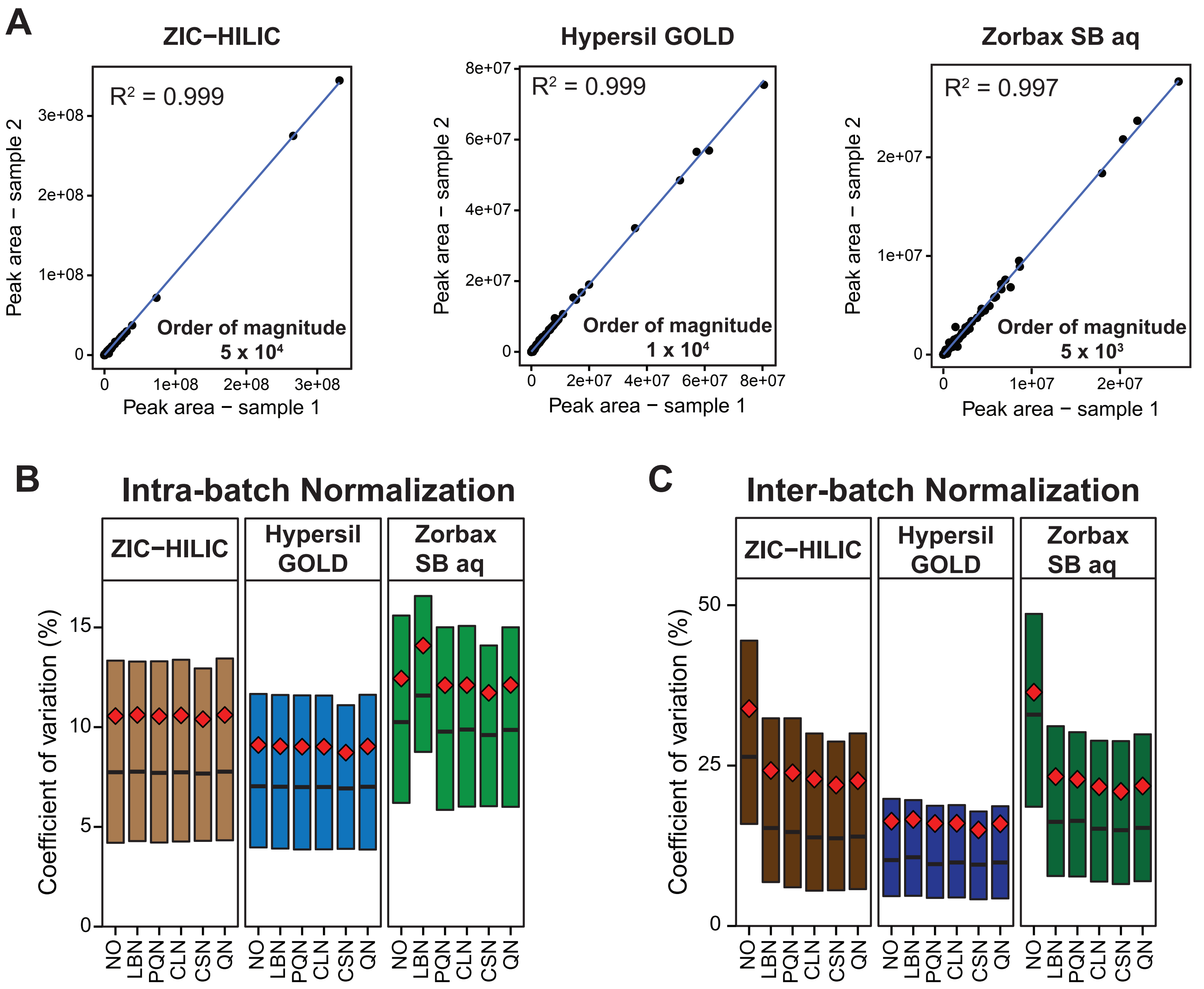
A
ZIC−HILIC
R^2 = 0.999
Order of magnitude
5 x 10^4
Peak area − sample 2
Peak area − sample 1
0
1e+08
2e+08
3e+08
Hypersil GOLD
R^2 = 0.999
Order of magnitude
1 x 10^4
2e+07
4e+07
6e+07
8e+07
Zorbax SB aq
R^2 = 0.997
Order of magnitude
5 x 10^3
1e+07
2e+07
B
Intra-batch Normalization
Coefficient of variation (%)
0
5
10
15
Hypersil GOLD
Zorbax SB aq
NO
LBN
PQN
CLN
CSN
QN
C
Inter-batch Normalization
25
50

Figure S8.

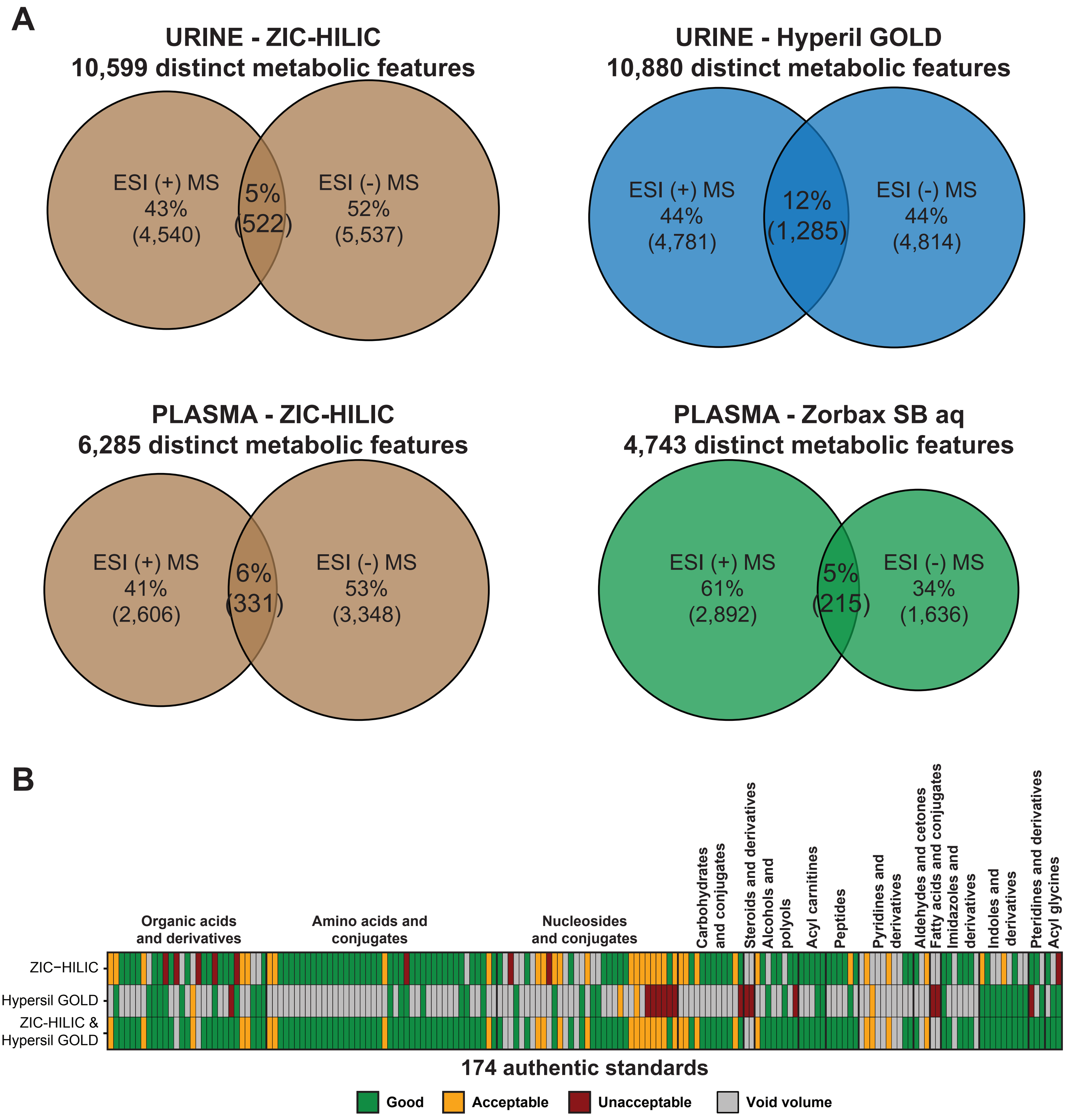
A
URINE - ZIC-HILIC
10,599 distinct metabolic features
ESI (+) MS
43%
(4,540)
5%
(522)
ESI (-) MS
52%
(5,537)
URINE - Hyperil GOLD
10,880 distinct metabolic features
ESI (+) MS
44%
(4,781)
12%
(1,285)
ESI (-) MS
44%
(4,814)
PLASMA - ZIC-HILIC
6,285 distinct metabolic features
ESI (+) MS
41%
(2,606)
6%
(331)
ESI (-) MS
53%
(3,348)
PLASMA - Zorbax SB aq
4,743 distinct metabolic features
ESI (+) MS
61%
(2,892)
5%
(215)
ESI (-) MS
34%
(1,636)
B
Organic acids and derivatives
Amino acids and conjugates
Nucleosides and conjugates
Carbohydrates and conjugates
Steroids and derivatives
Alcohols and polyols
Acyl carnitines
Peptides
Pyridines and derivatives
Aldehydes and cetones
Fatty acids and conjugates
Imidazoles and derivatives
Indoles and derivatives
Pteridines and derivatives
Acyl glycines
ZIC−HILIC
Hypersil GOLD
ZIC-HILIC & Hypersil GOLD
174 authentic standards
Good
Acceptable
Unacceptable
Void volume

Figure S9.

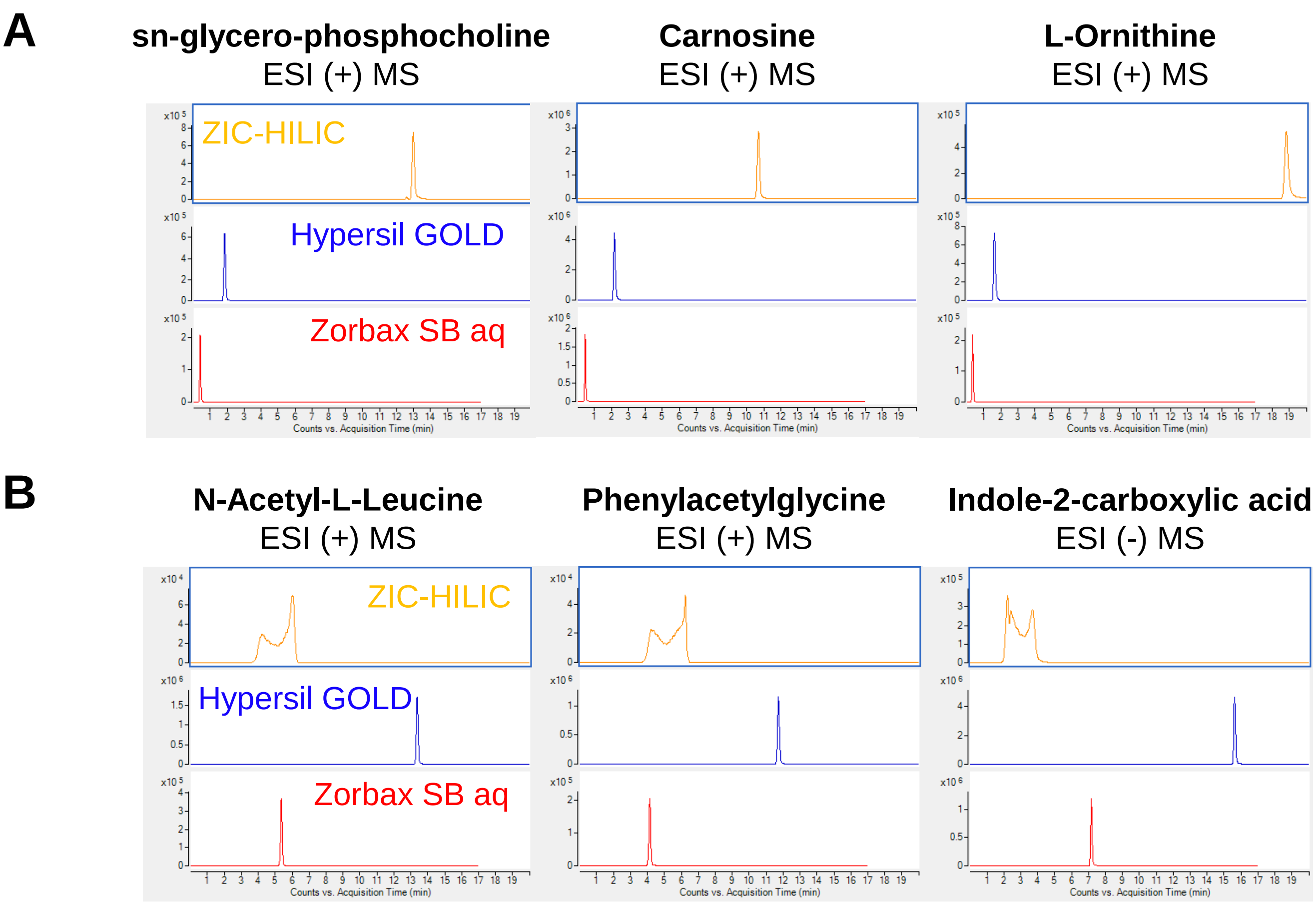
A
sn-glycero-phosphocholine
ESI (+) MS
Carnosine
ESI (+) MS
L-Ornithine
ESI (+) MS
ZIC-HILIC
Hypersil GOLD
Zorbax SB aq
Counts vs. Acquisition Time (min)
B
N-Acetyl-L-Leucine
ESI (+) MS
Phenylacetylglycine
ESI (+) MS
Indole-2-carboxylic acid
ESI (-) MS
ZIC-HILIC
Hypersil GOLD
Zorbax SB aq
Counts vs. Acquisition Time (min)
